# Systems Pharmacological Approach to Investigate the Mechanism of* Acori Tatarinowii Rhizoma* for Alzheimer's Disease

**DOI:** 10.1155/2018/5194016

**Published:** 2018-06-27

**Authors:** Zhenyan Song, Fang Yin, Biao Xiang, Bin Lan, Shaowu Cheng

**Affiliations:** The Key Laboratory of Hunan Province for Integrated Traditional Chinese and Western Medicine on Prevention and Treatment of Cardio-Cerebral Diseases, Hunan University of Chinese Medicine, Changsha, Hunan 410208, China

## Abstract

In traditional Chinese medicine (TCM),* Acori Tatarinowii Rhizoma* (*ATR*) is widely used to treat memory and cognition dysfunction. This study aimed to confirm evidence regarding the potential therapeutic effect of* ATR* on Alzheimer's disease (AD) using a system network level based* in silico* approach. Study results showed that the compounds in* ATR* are highly connected to AD-related signaling pathways, biological processes, and organs. These findings were confirmed by compound-target network, target-organ location network, gene ontology analysis, and KEGG pathway enrichment analysis. Most compounds in* ATR* have been reported to have antifibrillar amyloid plaques, anti-tau phosphorylation, and anti-inflammatory effects. Our results indicated that compounds in* ATR* interact with multiple targets in a synergetic way. Furthermore, the mRNA expressions of genes targeted by* ATR* are elevated significantly in heart, brain, and liver. Our results suggest that the anti-inflammatory and immune system enhancing effects of* ATR* might contribute to its major therapeutic effects on Alzheimer's disease.

## 1. Introduction

Alzheimer's disease (AD) is one of the most common age-related serious fatal neurodegenerative diseases, and its prominent feature is progressive cognitive, learning, and memory dysfunction. Gradually, bodily functions are lost, eventually leading to death [1]. According to a statistical report issued by Alzheimer's disease association in 2016, one new case of AD is expected to develop every 33 seconds, resulting in nearly 1 million new cases per year, and the estimated prevalence is expected to range from 11 million to 16 million by 2050 [2]. Therefore, prevention and treatment of AD are particularly important. Although AD is a global health problem, its pathology remains poorly understood [3]. About 70% of the risk is thought to be genetic, and many genes are usually involved. The disease process is associated with high density of senile plaques and neurofibrillary tangle in brain [4]. Currently, no drugs or supplements can reduce the processes [2].

Recent study shows that AD might result from a series of complex reasons, such as aging, immunity dysfunction, and metabolic disturbances [5, 6]. In order to understand the pathogenesis of complex diseases a new “multitarget, multidrug” model strategy of drug discovery was presented [7]. Interestingly, as a multidisciplinary system of multicomponent therapeutics, Traditional Chinese Medicine (TCM) Systems revealed a probability to explain the relationship between multicomponents and drug synergistic effects [8].* Acori Tatarinowii Rhizoma* (ShiChangPu,* ATR*) is a well-known herb from Chinese traditional medicine (TCM) being used for neurodegenerative diseases in China at least 2,000 years.* ATR* was recorded in the Chinese pharmacopoeia (2015) as the official botanical source. It contains several phytochemicals, and the main ingredients include *β*-asarone, *α*-asarone, estragole,* cis*-methylisoeugenol, isoshyobunone, *δ*-cadinene, and methyleugenol. Some experimental researches by cellular and animal models support that* ATR* had anti-AD effects. Volatile oils of* ATR* were being used in AD for promoting neural progenitor proliferation, protecting *β*-amyloid induced neurotoxicity, and improving memory and cognitive function [9–11]. *β*-Asarone, a major essential oil component from* ATR, *has anti-AD effects by nerve growth factor signaling pathways, ameliorating oxidative stress, autophagy, and neuronal apoptosis* in vivo *[12–14]. In addition, many studies had confirmed that* ATR* has therapeutic effects on nervous disorders, depression, epilepsy, and dementia [15–19].

In recent years, systems pharmacology has emerged as a new field that integrates pharmacology, biochemistry, genomics, and bioinformatics techniques, to establishe a model based on computer network analysis and therapeutic targets prediction, and it highlights the holistic thinking of TCM [20]. It provides a new powerful tool to analyze and visualize the complex interaction data in herb, compound, target, and disease. To introduce the TCM application on AD by using systems pharmacology approach, Cai's group used a systems pharmacology approach to reveal the underlying molecular mechanisms of BSYZ in treating AD [21]. Luo's group established multiple herbs-compounds-targets-pathway-cooperation networks model to analyze the effects of Danggui-Shaoyao-San in AD [22]. Fang et al. performed literature mining of PubMed for top 10 anti-AD herbs (include* ATR*), based on systematic pharmacological analysis, and they interpreted the multiscale mechanisms of action of herbs in AD management [23]. Although these researches provided valuable support for the mechanisms of TCM in the treatment of AD, there is no analysis on the mechanism of the single herb, such as* ATR*, because they were based on the combined action of multiple herbs.

**Figure 1:**
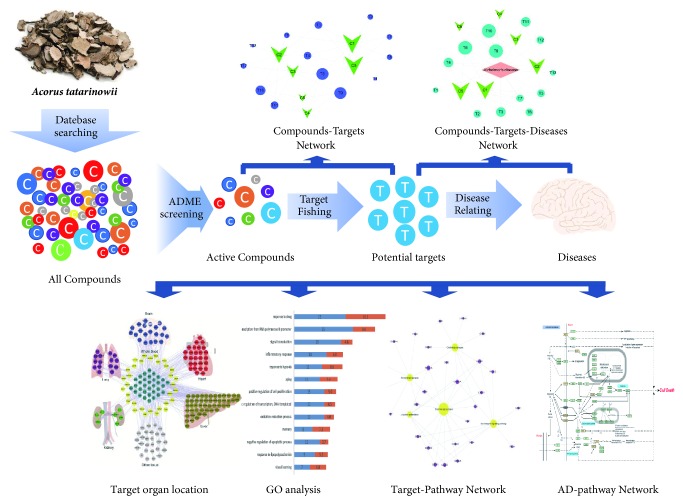
Workflow of system pharmacology by* Acori Tatarinowii Rhizoma* (*ATR*): screening and target fishing, network analysis, relevant organ location network analysis, gene ontology, and KEGG pathway analysis.

In this study, we discussed how system pharmacology demonstrated the relationship of* ATR* to treat AD from the system level. First, ingredients of ATR were collected from traditional Chinese medicine systems pharmacology (TCMSP, http://lsp.nwu.edu.cn/) database [24]. Oral bioavailability (OB), drug-likeness (DL), and blood-brain barrier (BBB) analysis were carried out to filter the compounds for subsequent analysis. Next, system pharmacology approach was used to explore the compounds-targets interactions and to establish the compounds-targets network and the compounds-targets-AD network. Finally, based on bioinformatics analysis, we illuminated the multiscale mechanisms of action of ATR on AD. A workflow of the systems pharmacological study was summarized as shown in Figure 1.

## 2. Material and Methods

### 2.1. Establishment of Database

All the compounds of* ATR* are collected by TCMSP database (a free phytochemical database of herbal medicine). Evaluating the absorption, distribution, metabolism, and excretion (ADME) parameters: oral bioavailability (OB), drug-likeness (DL), and blood-brain barrier (BBB) for all the natural compounds, the active compounds were collected to illustrate the proposed model.

### 2.2. OB Evaluation

OB shows the percentage of an active compound that reaches the systemic circulation, which defines one of the most important pharmacokinetic parameters to the convergence of the ADME process [25]. OB is essential to determine whether the chemical components of TCM have pharmacodynamic activity. The OB values are predicted by using the OBioavail 1.1 [26], which is a tool that contains 805 different structures of drugs and drug-like molecules and metabolism information. In this study, the OB threshold was set as ≥15%.

### 2.3. DL Prediction

DL helps filter out “drug-like" compounds in the traditional Chinese herbs, as DL is a qualitative concept used in drug design for an estimate on how “drug-like” a prospective compound is [27]. Compounds with high DL values are more likely to have certain biological properties that can increase the possibility for drug treatment. The calculation of DL value is based on Tanimoto coefficient formula in TCMSP database [28].

### 2.4. BBB Screening

The blood-brain barrier (BBB) is anatomically characterized by the presence of intercellular tight junctions between continuous nonfenestrated endothelial cells, whose normal function is to limit the passage of protein, potentially diagnostic and therapeutic agents into the brain parenchyma [29]. Understanding and evaluating the capacity of compounds of entering into the central nervous system, the compounds with BBB <-0.3 were considered as nonpenetrating (BBB-), those from -0.3 to +0.3 moderate penetrating (BBB±), and those >0.3 strong penetrating (BBB+) [8].

### 2.5. Target Fishing

To obtain active compounds of* ATR* target information, a comprehensive drug targeting protocol includes text mining database search and chemometric analysis was applied. First, the information on molecular targets was found from the TCMSP. Second, based on the set-wise chemical similarity among their ligands of active compounds, the targets were adopted by Similarity Ensemble Approach (SEA, http://sea.bkslab.org) [30] and the Binding Database (http://www.bindingdb.org) [31]. Third, the targets obtained from different databases were input to UniProt (http://www.uniprot.org/) [32] to make the targets names uniformly standardized. Finally, noise and error information was eliminated by PharGKB (https://www.pharmgkb.org/) [33], Therapeutic Target Database (DTT, http://bidd.nus.edu.sg/group/cjttd/) [34], and the Comparative Toxicogenomics Database (CTD, http://ctdbase.org/) [35].

### 2.6. Drug-Target Network

In order to understand the complex relationship between* ATR* active compounds and potential targets, a visual network is established by CytoScape_v3.4.0 [36]. This network is composed of node and edge. Nodes represent to molecules (compounds and targets), and edges indicate intermolecular interactions (compounds and targets interactions), namely, the connections between nodes.

### 2.7. Drug-Target-AD Network

DisGeNET database (http://www.disgenet.org/) [37] is a discovery platform that serves as one of the largest publicly available databases, containing a mass of collections of genes and variants associated with human diseases. It contains 561,119 gene-disease associations and 135,588 variant-disease associations. Based on expert curated repositories, GWAS catalogues, animal models, and the scientific literature, it can be used for different research purposes, including the generation of hypothesis on drug therapeutic action and drug adverse effects. In order to elucidate the action relations of* ATR* in Alzheimer's disease, the integration is performed by means of gene and AD vocabulary mapping and using the DisGeNET association type ontology to select the targets related to AD. The active compounds-targets-AD network is established by CytoScape_v3.4.0.

### 2.8. Target-Organ Location Network

BioGPS is a database for querying and organizing genetic annotation resources [38]. It provides gene expression abundance data in cells or tissues by microarrays analysis [39]. The target-organ location network is constructed with the use of the dataset: GeneAtlas U133A, gcrma (http://biogps.org). First, the mRNA expression patterns of each target gene are obtained in 84 parts of organ tissues. Second, average values for each gene are applied to computer. Third, genes will locate in the relevant organ tissues where the mRNA expression level is higher than the mean. Finally, a target-organ location network is constructed using Cytoscape 3.4.0 and organ-specific, Alzheimer's disease related gene overexpression data.

### 2.9. Gene Ontology (GO) Analysis

Gene Ontology (GO) is a framework for the model of biology. The GO defines gene classes that describe gene functions and relationships between these concepts. It covers three domains: Cellular Component (CC) explains where gene products are active; Molecular Function (MF) means molecular activities of gene products; Biological Process (BP) interprets the pathways and larger processes made up of the activities of multiple gene products [40]. In this study, GO terms with* P* values < 0.01 and Benjamini < 0.05 were employed and the data were collected by the DAVID 6.8 Gene Functional Classification Tool [41] (http://david.abcc.ncifcrf.gov/).

### 2.10. Network Pathway Analysis

KEGG pathway enrichment analysis provides not only pathway functional annotations of given gene set but also pathway enrichment analysis [42]. First, gene numbers of each pathway in the given gene set are calculated. Then, significantly enriched pathways in the given gene set compared to the genome background are defined by hypergeometric test. The calculating formula of* P* value is

(1) $$\begin{array}{c} P=1-\overset{m-1}{\underset{i=0}{\sum }}\frac{\left(\begin{array}{c} M\\ i \end{array}\right)\left(\begin{array}{c} N-M\\ n-i \end{array}\right)}{\left(\begin{array}{c} N\\ n \end{array}\right)}. \end{array}$$

Here N is the number of all genes with KEGG pathway annotation (http://www.genome.jp/kegg/) [43] which represents the gene number of given genes set in N; M is the number of all genes that are annotated to the certain pathway; m is the gene number in the given gene set that is annotated to the certain pathways. Taking* P* values < 0.01 and FDR ≤ 0.05 as threshold, pathways that meet this condition were defined as significantly enriched pathways in the given genes set. In this research, pathway enrichment analysis was performed by using the OmicShare tools, a free online platform for data analysis (http://www.omicshare.com/tools/). In order to illuminate the action mechanisms of* ATR* in Alzheimer's disease, filtered target proteins were entered into the pathway map of Alzheimer's disease acquired from the KEGG.

## 3. Results

### 3.1. Identification of Active Compounds

As 105 compounds were identified belonging to* ATR* in TCMSP (as shown in Table S1), 30 compounds were selected with the conditions of OB≥15%, BBB≥-0.3, and DL≥0.1. Removing 2 compounds (4-aromadendrene, isocembrol) with no target information, 28 compounds with known target information were chosen for the following analysis. Another 4 compounds (*β*-asarone, *γ*-asarone, cis-methylisoeugenol, and methyleugenol) reported to have anti-inflammation, antioxidation, and antimicrobial effects were added additionally [13, 44–46], and finally 32 compounds were analyzed as shown in Table 1.

Most of these 32 compounds showed various biological activities experimentally. For example, eudesmin (M23; DL=0.62, BBB=0.05, and OB=52.35) was experimentally identified which has anti-inflammation [47] and anticonvulsion effects [48]. A angiogenesis inhibitor of marmesin (M28; DL=0.18, BBB=0.07, and OB=50.28) was reported to have regulatory effect on endothelial cell fate and angiogenesis [49]. Antioxidation and anti-inflammation effect of patchoulene (M31; DL=0.11, BBB=2.17, and OB=49.06) was proved through inhibition of nuclear factor *κ*B (NF-*κ*B) and downregulation of COX-2/iNOS [50]. Bergapten (M12; DL=0.13, BBB=0.69, and OB=42.21) was reported to have anti-inflammatory properties [51] and the ability of ameliorating the insulin resistance [52]. Aminacrine (M9; DL=0.12, BBB=0.33, and OB=35.00) and its derivatives were experimentally identified to be reversible inhibitors of acetyl cholinesterase (AChE) [53]. *α*-Cedrene (M2; DL=0.10, BBB=2.16, and OB=55.56) was reported to exhibit antimicrobial activity [54]. Veraguensin (M7; DL=0.39, BBB=0.72, and OB=25.53) was reported to have analgesic and anti-inflammatory activities [55] and neuroprotective effects[56]. *β*-Asarone (M16; DL=0.06, BBB=1.24, and OB=35.61) increased striatal level of dopamine by enhancing dopa decarboxylase activity which improved behavioral competence in Parkinson's rat model [57]. *γ*-Asarone (M24; DL=0.06, BBB=1.33, and OB=22.76) prevented oxidative stress-induced cell injury in cultured astrocytes through Akt signaling activation [46]. Aristolone (M11; DL=0.13, BBB=1.54, and OB=45.31) was reported to have regulatory effects on lipid peroxidation and proliferation in human cancer cells [58]. *α*-Cubebene (M10; DL=0.11, BBB=2.1, and OB=16.73) was experimentally identified with significant antifungal activity inhibition of* A. flavus* growth [59]. *β*-Gurjunene (M15; DL=0.10, BBB=2.07, and OB=51.36) was experimentally identified with antioxidation activity and antimicrobial activity [60]. Isopimpinellin (M26; DL=0.17, BBB=0.5, and OB=25.93) was reported to exhibit antiallergic inflammation [61]. *α*-Longipinene (M3; DL=0.12, BBB=2.05, and OB=57.47) was reported to inhibit biofilm formation and hyphal growth in* Candida albicans* [62]. Anti-inflammation effect of aristolene (M11; DL=0.11, BBB=2.08, and OB=52.2) was proved through measuring secretion of the proinflammatory cytokines IL-6 and TNF-*α* by using a human-derived macrophage cell line [63]. Calarene (M20; DL=0.11, BBB=2.04, and OB=52.16) and caryophyllene oxide (M4; DL=0.13, BBB=1.76, and OB=32.67) from the essential oil of* Patrinia scabiosaefolia* were both found to have antineuroinflammation, anticancer, and antioxidation effects[64]. As mentioned above, many compounds of* ATR*, which are universal in herbs, plants, and fruits, contribute to neuroprotective, anti-inflammation, antimicrobial, and antioxidation effects.

### 3.2. Target Fishing

These 32 identified active compounds interact with 181 target proteins (as shown in Table S2) based on a target fishing technique [65]; that is, on average, they interact with 5.7 target genes, which did fully explain the multiple-target effects of pharmacology by* ATR *[66]. Among the 181 obtained targets entered to DisGeNET database for screening AD-related targets, only 97 potential targets (as shown in Table S3) were associated with AD, and they were reserved for further analysis.

### 3.3. Network Construction

To facilitate the visualization of multiple-target effects of* ATR* and AD interrelation, network analysis was performed based on the context of the whole human genome [67]. Two networks (A) C-T and (B) C-T-AD were constructed that might show multicompound and multitarget effects and they might explain why* ATR* can be used for the treatment of AD.

#### 3.3.1. Compound-Target Network (C-T Network)

**Figure 2:**
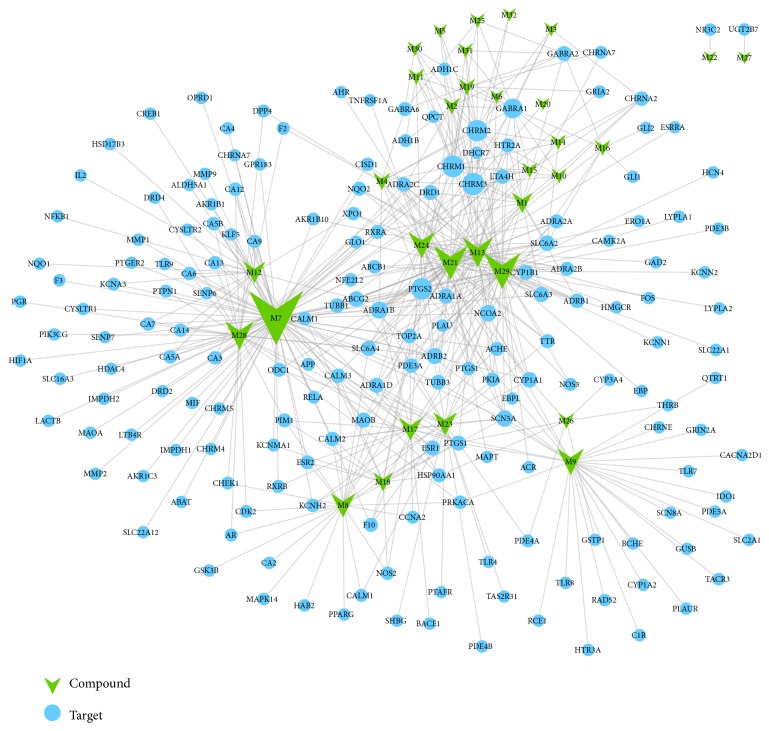
C-T network: a compound-target (C-T) network and nodes represent compounds and targets.

C-T interactions data between 181 targets and 32 compounds of* ATR* were collected by the method in Section 2.5. The network comprised 213 nodes and 509 edges. Circular nodes represented targets and triangle nodes represented compounds. Node size was regulated by degree centrality, resulting in an average degree of 4.70 nodes per target and 15.91 edges per compound, respectively (as shown in Figure 2). Among the 32 active compounds, 10 compounds with high-degree distributions were identified, and each of these compounds hit more than 21 potential targets. Usually, higher degree meant that the compounds had more important pharmacological effects on the potential treatment of AD [68]. For example, veraguensin (M7, degree =82) had the highest number of interactions with targets, followed by methyleugenol (M29, degree =47) and cis-methylisoeugenol (M21, degree = 43), indicating that those compounds might have more pharmacological effects than other compounds in this C-T network. Meanwhile, muscarinic acetylcholine receptor M3 (CHRM3, degree =20) showed the most intimate connections with compounds, followed by muscarinic acetylcholine receptor M1 (CHRM1, degree = 19) and prostaglandin G/H synthase 2 (PTGS2, degree=18). The C-T network results demonstrated the multitarget effects of active compounds by* ATR*.

#### 3.3.2. Compound-Target-AD Network (C-T-AD Network)

**Figure 3:**
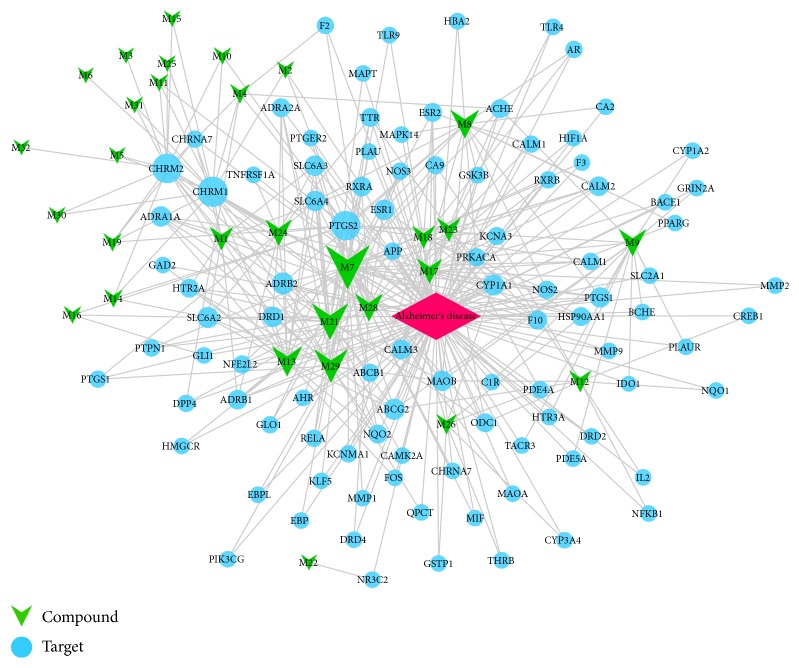
C-T-AD network: a compound-target-Alzheimer's disease (C-T-AD) network and nodes represent compounds and targets.

Network pharmacological methods provided a visual approach to understanding the complex relationship between disease and therapeutic spots [69, 70]. In the present study, 97 potential targets of AD were selected form DisGeNET database and 30 involved compounds (M27 and M30 have no target) were used to construct the C-T-AD network for further cluster analysis. The C-T-AD network was built in the same way as C-T network, as shown in Figure 3. Veraguensin (M7, degree=42), cis-methylisoeugenol (M21, degree=28), methyleugenol (M29, degree =24), and *β*-asarone (M13, degree=20) might play an important role in the treatment of AD. CHRM1 (degree = 20), PTGS2 (degree = 19), CHRM2 (degree = 19), and ADRB2 (degree = 9) might be the important drug-targets. 26 active compounds connected with more than two targets and all of the 97 targets interact with more than one compound, indicating that many proteins associated with AD might display similar binding patterns with ligands.

### 3.4. Multiple*-*Target/Organ Cooperation for AD Treatment

Traditional Chinese medical workers consider the human body as an organic whole, and none of the organs in this whole organization are independent. Theses organs are structurally inseparable, functionally coordinated, and complementary to each other. They interact with each other in pathology. Therefore, in this study, the complex process of exploring* ATR* for AD was used by multiorgan cooperation and multitarget action.

#### 3.4.1. GO Analysis

**Figure 4:**
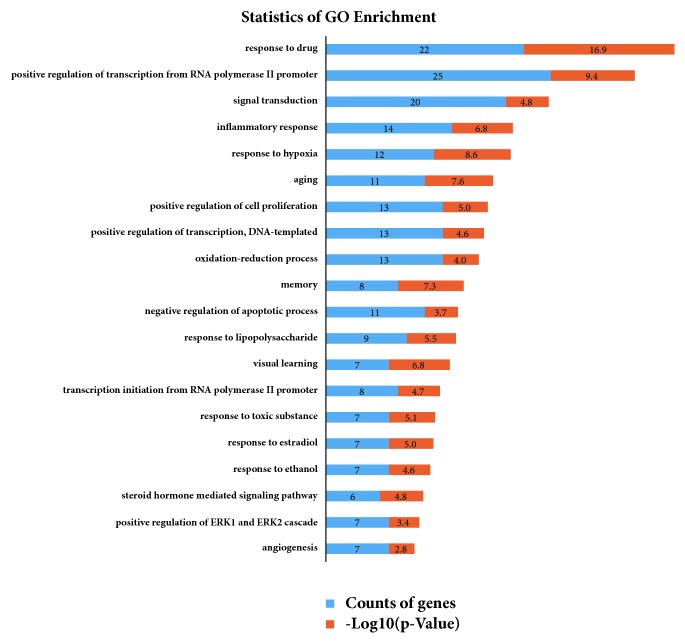
GO analysis: 20 biological processes (BP) of gene ontology (GO) terms sorted by* P *value < 0.01. Counts of genes and* P *value related to each BP terms are shown. The* x*-axis represents BP terms for the target genes, and the* y*-axis shows counts of genes and −log10 (*P *value).

GOBP describes a series of events accomplished by one or more organized assemblies of molecular functions [40]. GOBP showed that these targets were enriched to 20 biological process terms, and the 181 genes were highly related to response to drug, signal transduction, inflammatory response, response to hypoxia, aging, memory, and so on (as shown in Figure 4). In detail, phosphatidylinositol-4, 5-bisphosphate 3-kinase catalytic subunit, gamma isoform (PIK3CG), toll-like receptor 4 (TLR4), tumor necrosis factor receptor superfamily member 1A (TNFRSF1A), protooncogene c-fos (FOS), nuclear factor erythroid 2-related factor 2 (NFE2L2), macrophage migration inhibitory factor (MIF), and nuclear factor NF-kappa-B p105 subunit (NFKB1) were involved. 14 genes were related to “inflammatory response,” including sodium-dependent serotonin transporter (SLC6A4), matrix metalloproteinase-2 (MMP-2), hypoxia-inducible factor 1-alpha (HIF1A), dopamine D2 receptor (DRD2), inducible nitric oxide synthase (NOS2), and cyclic AMP-responsive element-binding protein 1 (CREB1). 12 genes were related to “response to hypoxia,” such as NFE2L2, Alpha-1A adrenergic receptor (ADRA1A), neuromedin-K receptor (TACR3), 5-hydroxytryptamine 2A receptor (HTR2A), transcription factor p65 (RELA), 3-hydroxy-3-methylglutaryl-coenzyme A reductase (HMGCR), sodium-dependent dopamine transporter (SLC6A3), FOS, and CREB1. 11 genes were related to “aging” including SLC6A4, HTR2A, PTGS2, DRD1, CREB1, neuronal acetylcholine receptor subunit alpha-7 (CHRNA7), glutamate receptor ionotropic, and NMDA 2A (GRIN2A). 11 genes were related to “memory.”

In conclusion,* ATR* is entirely possible as a therapeutical herb on AD, which is attributed to affect certain biological processes, such as aging, memory, anti-inflammation, and response to hypoxia.

#### 3.4.2. Target Tissue Location

The mRNA specific expression patterns in tissues are important clues to the gene function [71]. Thus, they are of great significance to determine the tissue mRNA expression profiles of various genes at the organ level which is to explore the beneficial effects of* ATR* on AD [72]. In this paper, the results of mRNA expression were demonstrated by using BioGPS microarray analysis. 175 of the 181 targets had their expression profiles in 84 normal tissues (as shown in Table S3).

**Figure 5:**
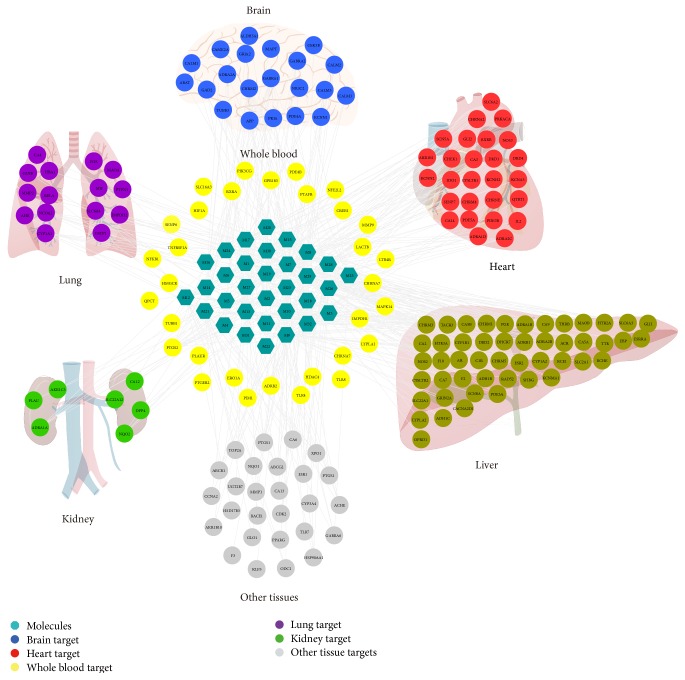
Target-organ location map. The node pie chart represents the organs in which each target is located.

The 175 targets were generally found in human tissues but showed different levels of mRNA expression. We focused on some of these organizations, including brain, heart, kidney, liver, lung, and whole blood. A target was positioned to the organization where it had the highest mRNA expression level. For example, we compared the expression patterns of different tissues and observed that the 21 targets contained had higher mRNA expression levels in the brain than the remaining 84 normal tissues, 27 targets were positioned in heart, and, meanwhile, 32 targets located in whole blood were linked with tissues in any forms. The organization distribution network of 175 targets (as shown in Figure 5) is based on their expression patterns.

Results from our research suggested that the 21 high-abundant targets in brain were regarded as therapeutic targets for AD, and the other targets were also thought to be associated with AD. Our results implied that drug targets worked on these tissues rather than focusing on a specific one, and the whole blood serves as bridge between these tissues, so as to promote the coordination of organs to achieve the positive effect of AD. The effects of AD are not just in the brain but also in other organs. For example, AD can cause a series of bodily functions damage incidents such as cardiac autonomic dysfunction, urinary incontinence, and metabolic disorders [2, 73, 74]. Meanwhile, other organ lesions may also affect the progression of AD. For example, insulin resistance, hyperglycemia, hyperinsulinemia, and type 2 diabetes mellitus could provoke AD [75]. Coronary heart disease and heart surgery correlate with the development of AD cognitive dysfunction [76, 77]. Therefore, the prevention of AD requires not only attention to the brain but also the coordination of other organs. An interpretation of these findings is that the holistic view of TCMs diagnosis and treatment theory is correct.

#### 3.4.3. Alzheimer's Disease-Relating Pathway

**Figure 6:**
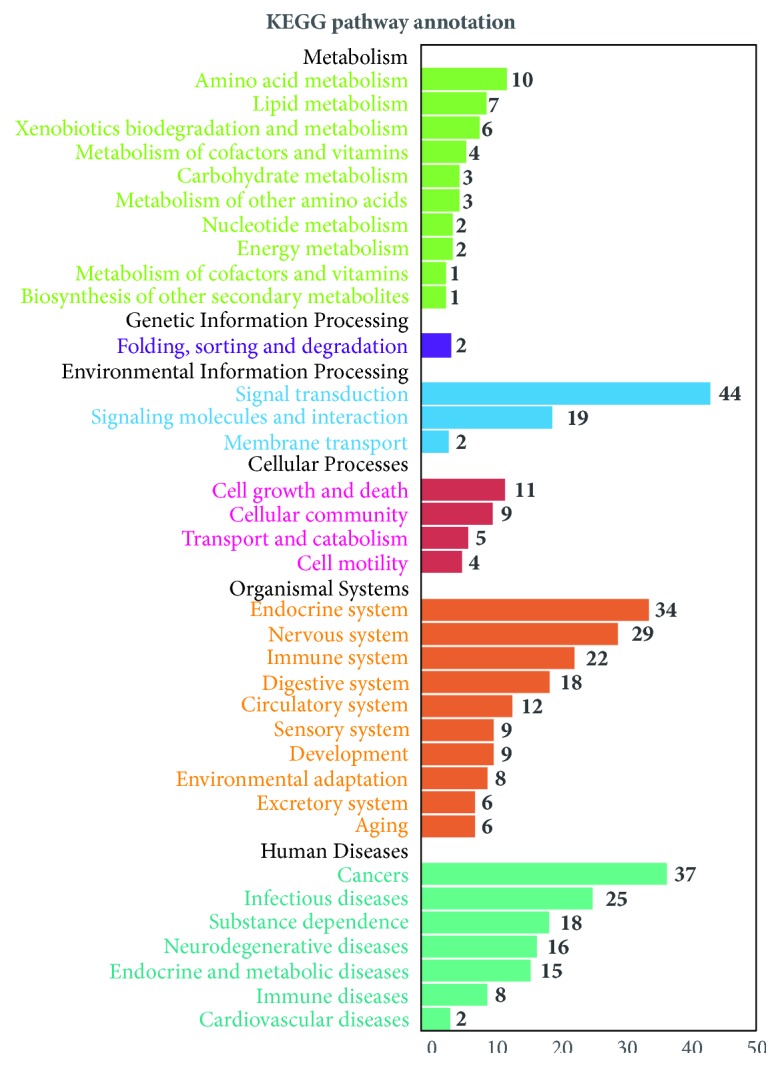
KEGG pathway annotation.* x*-axis represents B level classification of pathways, and the* y*-axis shows the gene number in the given gene set that are annotated to the certain pathways.

Through the C-T-AD network, a macroscopic visual of the relationship between* ATR*, targets, and AD was obtained, but the detailed mechanism of these remains unclear. Therefore, for determining the interactions among ATR and AD multiple targets, we did the following things. First, 97 potential targets of AD underwent pathway enrichment analysis, and a holistic picture of KEGG pathway annotation constructed by the use of the OmicShare tools was provided (as shown in Figure 6). Second, an “AD-pathway” was established based on Alzheimer's disease pathway which was provided by the KEGG pathway database (as shown in Figure 9). 

*(1) KEGG Pathway Annotation*. KEGG pathway annotation can be divided into several modules, such as “Environmental Information Processing”, “organismal systems”, and “metabolism.” Two representative modules, “environmental information processing” and “organismal systems,” were described in detail to clarify the underlying mechanism.

**Figure 7:**
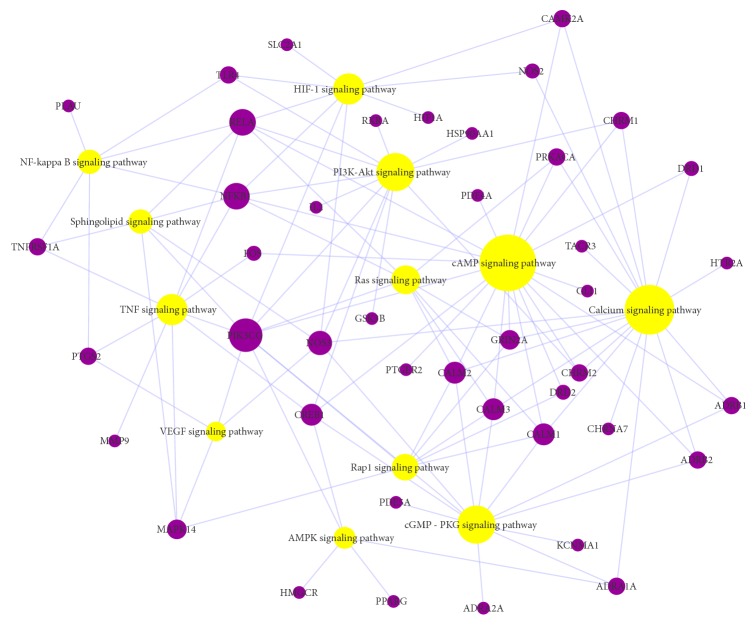
A target-pathway network (T-P) was displayed, signal transduction in environmental information processing.

Signal transduction in “environmental information processing” module was studied, and a target-pathway network (T-P) was displayed (as shown in Figure 7) whose node size was regulated by degree centrality. 12 signal transduction pathways were concentrated.

There were 4 inflammation-related pathways shown in signal transduction module, including TNF signaling pathway (degree=9), NF-kappa B signaling pathway (degree=6), HIF-1 signaling pathway (degree=9), and VEGF signaling pathway (degree=4), and these pathways indicated that the anti-inflammatory action is important for the treatment of AD. The synthesis of inflammatory critical cytokines and receptors could induce a wide range of intracellular biological processes including apoptosis and cell survival as well as inflammation and immunity [78, 79]. We found that *γ*-asarone (M24) targets TNF receptor superfamily member 1A (TNFRSF1A), cis-methylisoeugenol (M21) and veraguensin (M7) target NF-kappa B p65 subunit (RELA), bergapten (M12) targets NF-kappa B p105 subunit (NFKB1), and eudesmin (M23) targets toll-like receptor 4 (TLR4), and so on.

The G-protein-coupled receptor-triggered signaling cascade related pathways about cAMP signaling pathway (degree=20) had the maximum interaction targets, suggesting that it was closely related to AD. Research shows that activation of cAMP-dependent pathway may lead to phosphorylate alpha-amino-3-hydroxy-5-methyl-4-isoxazolepropionic acid (AMPA) [80], tau hyperphosphorylation, and memory impairment [81].

Calcium signaling pathway also plays an important role (degree=17). Calcium can act in signal transduction resulting from activation of ion channels or as a second messenger caused by indirect signal transduction pathways such as G-protein-coupled receptors [82]. In neurons, calcium is important for the synchronization of neuronal electrical activity with mitochondrial energy metabolism when it increases in cytosolic and mitochondrial system [83], and the increasing of Ca^2+^ signals due to Ca^2+^ release has been implicated to play roles in synaptic plasticity and memory, neurotransmitter release, and neuronal excitability[84].

The signaling pathway mediated by small GTPase affects the structure and function of synapses, such as Ras signaling pathway (degree=8) and Rap1 signaling pathway (degree=7). For example, Ras protein family (Ras, Rap1, and Rap2), which belong to small GTPase, regulate the secretion of presynaptic neurotransmitters and the transshipment of the postsynaptic glutamate receptors in the cell membrane [85].

PI3K-Akt signaling pathway (degree=12) is important to regulate the cell cycle and promote growth and proliferation neural stem cells (NSCs) differentiate into motor neurons in adult [86] and promote neuroprotection from NSCs injury [87]. 2′-O-Methylisoliquiritigenin (M8) targets glycogen synthase kinase-3 beta (GSK3*β*). GSK3*β* plays an important role in the ultraphosphorylation of tau, which is one of the pathological features of AD. Increased GSK3*β* activity causes PI3K/Akt dysfunction, which regulates glucose metabolism in the brain and leads to tau hyperphosphorylation in the brain of AD patients [88]. Moreover, in long-term potentiation (LTP), the PI3K binds to an AMPA receptor in a conservative region to determine the receptor in the membrane, especially in the GluR subunit, which causes AMPA receptors to be inserted postsynaptically [89].

In addition, cGMP-PKG signaling pathway (degree=12), sphingolipid signaling pathway (degree=6), and AMPK signaling pathway (degree=5) are also thought to be related to AD.

**Figure 8:**
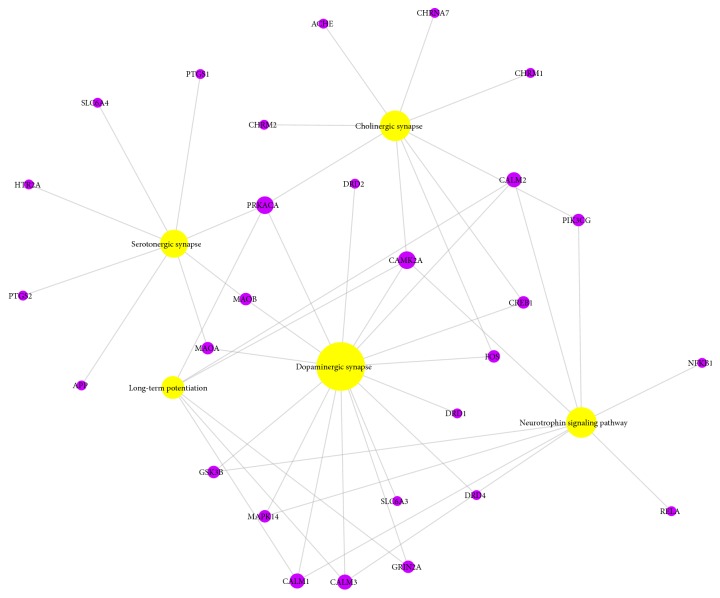
A target-pathway network (T-P) was displayed, nervous system in organismal systems module.

Nervous system in organismal systems module was also studied, and a target-pathway network (T-P) was established (as shown in Figure 8). Node size was regulated by degree centrality. They display extremely significantly close functional correlation to the AD, including dopaminergic synapse, cholinergic synapse, neurotrophin signaling pathway, serotonergic synapse, and long-term potentiation (LTP). Some researches showed that AD was also recognized as a disease of synaptic failure. Beta-secretase 1 (BACE1) cleaved amyloid precursor protein (APP) products impact learning and memory through proteins localized on glutamatergic, GABAergic, and dopaminergic synapses [90]. Acetylcholine is combined with both muscarinic acetylcholine receptor M1 (CHRM1) and muscarinic acetylcholine receptor M2 (CHRM2). To protect the memory and attention deficit caused by the loss of cholinergic neurons [91], acetylcholinesterase (ACHE) inhibitor works by partially blocking the degradation of acetylcholine in the synapse and enabling more of the neurotransmitter to reach and activate cholinergic receptors [92]. Soluble oligomers of the amyloid-*β* peptide (A*β*O) decreased brain serotonin (5-HT) levels in mice, whereas treatment with 5-HT prevented A*β*O-induced microglial activation and increased TNF-*α* levels [93]. We found that bisasarcin (M17) targets BACE1 and ACHE. (-)-Caryophyllene oxide (M4), veraguensin (M7), and aminacrine (M9) target ACHE. Among the 32 identified active compounds, 19 compounds target CHRM1 and 18 compounds target CHRM2, respectively. (-)-Alloaromadendrene (M1), *β*-asarone (M13), cis-methylisoeugenol (M21), and methyleugenol (M29) target 5-hydroxytryptamine 2A receptor (HTR2A).

**Figure 9:**
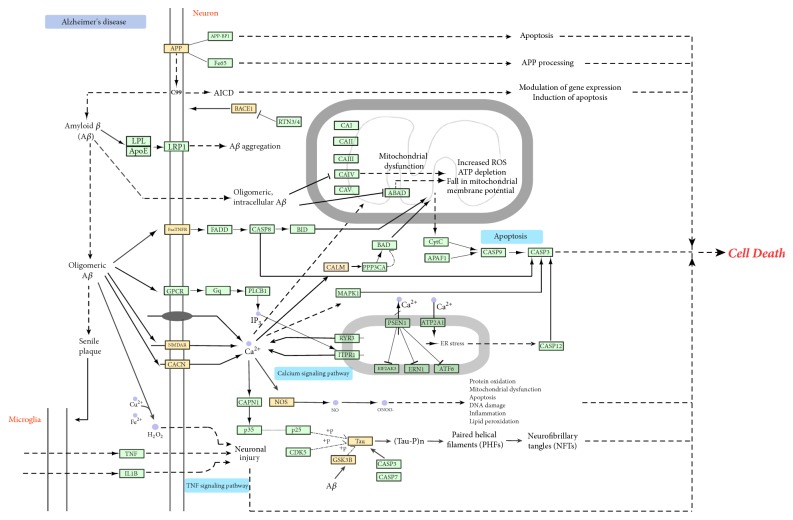
Network pathway: pathway enrichment analysis was performed. 97 filtered target genes were mapped into Alzheimer's disease pathway from the Kyoto Encyclopedia of Genes and Genomes (KEGG) to confirm the possible effect pathway of* Acori Tatarinowii Rhizoma* (*ATR*) on Alzheimer's disease.

*(2) AD-Pathway Network*. Nine protein targets were mapped to the AD-pathway network, indicating that these proteins play an important role in AD (as shown in Figure 9). AD is associated with senile plaques and neurofibrillary tangles (NFTs). Amyloid-beta (A*β*), a major component of senile plaques, has various pathological effects on cell and organelle function. Intracellular A*β* may contribute to pathology by facilitating tau hyperphosphorylation, disrupting mitochondria function, and triggering calcium dysfunction. As displayed in Figure 9. Some target receptors involved in A*β* aggregation and tau hyperphosphorylation were affected by* ATR* components to produce neuroprotective functions.

Recent studies have shown that the soluble oligomers of A*β* polypeptides may be a pathogenic factor for the development of Alzheimer's disease [94]. The amyloidogenic pathway triggered from two sequential cleavages of APP by BACE1 resulted in the formation of A*β* peptides [95]. Based on the above findings, blocking the enzyme (BACE inhibitor) in theory would prevent the accumulation of A*β* and (per the amyloid hypothesis) may help slow or stop Alzheimer's disease. For example, Albany Molecular Research Inc. reported on their development of an experimental drug for the treatment of Alzheimer's disease called verubecestat (MK-8931), which is an inhibitor of BACE1[96]. AstraZeneca and Eli Lilly and Company announced an agreement to codevelop lanabecestat (AZD3293), which is an oral beta-secretase 1 cleaving enzyme (BACE) inhibitor [97], and the drug has been developed with the FDA's fast-track designation. Our research data indicated that veraguensin (M7) and cis-methylisoeugenol (M21) target APP and bisasarcin (M17) can target BACE1 being used to treat AD through decreasing production of amyloid.

Excessive or abnormal tau phosphorylation destroys the cytoplasm function and interferes with axonal transport, resulting in cell death [98]. GSK3*β* has a great influence on activated overphosphorylation factor. 2′-O-Methylisoliquiritigenin (M8) targets GSK3*β* with AD treatment by preventing tau overphosphorylation.

As far as we know, glutamate-mediated excitotoxicity is one of the major reasons for chronic neurodegeneration. Aminacrine (M9) targets GRIN2A (NMDAR), and calamendiol (M19) and *γ*-asarone (M24) have shown connections with GRIA2 (GLuR). GRIA2 is a mediated glutamate receptor, which can mediate excitatory synaptic transmission in synaptic hyperactivity [99]. NMDA subtypes of glutamate receptors are agents that rely on the death of cells dependent on Ca^2+^ [100], which can lead to degenerative disorders due to excessive or inappropriate activity [101]. Therefore, GRIN2A and GRIA2 are the intrinsic neural protection targets for regulating Ca^2+^ concentration. Meanwhile, aminacrine (M9) targets voltage-dependent calcium channel subunit alpha-2/delta-1 (CACNA2D1), a protein in a voltage-dependent calcium channel mediates calcium ions into the membrane polarization. GRIN2B and GRM5 are associated with calcified neuropathy. Therefore, neuronal Ca^2+^ signaling is partially targeted through* ATR* for the treatment of AD.

In addition, TNFRSF1A (TNFR) participated in the MAPK signaling pathway and has been associated with inflammation, which is a risk factor for AD.

## 4. Discussion and Conclusion

TCM is a complex system composed of multicomponent and multitarget, and the function of each component is synergistic. The composition of TCM is so complex that it is difficult to study its effects from the mixture and to elaborate the mechanism of action from the perspective of modern medicine. The systematic pharmacological method provides new ideas and perspectives for the research of complicated Chinese medicine system. The pharmacology of TCM system has established a model of interaction of elements, such as drug molecules, protein targets, tissues, and organs, to clarify and predict the efficacy and toxicity of TCM [102]. In this study, a systematic pharmacological method was used to study the active ingredients of* ATR*. The potential targets of pharmacodynamic components, gene ontology, network analysis, organ location network, and pathway enrichment analysis were employed to explore the relationship between active ingredients and targets of Alzheimer's disease. Our result showed that* ATR* and AD are highly correlated in biological processes, organs, and signaling pathways. Our main findings are as follows.

(1) Through defining components in this system, we have studied systematic pharmacological methods to integrate multiple technologies, including ADME-system assessment, drug targeting, and target tissue distribution. 32 active compounds were detected. These compounds interact with 181 different targets by drug targeting. The resulting C-T network indicated that compounds veraguensin (M7), methyleugenol (M29), and cis-methylisoeugenol (M21) and targets CHRM3, CHRM1, and PTGS2 are the key factors to play an important role in the drug-target interaction network.

(2) The identified 97 targets associated with Alzheimer's disease are essential to understanding the pharmacological mechanisms of* ATR*. The C-T-AD network has clearly elucidated the pharmacological action and mechanism of* ATR* on Alzheimer's disease. For example, aristolone (M11) mainly targets at acetylcholine receptors CHRM1 and CHRM2 to treat AD through the cholinergic synapse pathway. The resulting network builds a more comprehensive visualization of the C-T-AD interaction pattern.

(3) TCM is the embodiment of holistic medicine, which is characterized as multicomponent treatment, multiobjective/approach regulation, and multiorgan cooperation. The response to inflammatory, hypoxia, aging, and memory mechanism of* ATR* is also illustrated by GO analysis. For instance, the 14 potential targets from the 7 compounds were enriched to the biological process of inflammatory response, which indicated that anti-inflammation may be the key to* ATR* treatment of AD. The target tissue location results had shown that 27 and 48 of 185 targets of* ATR* were highly expressed in heart and liver, respectively. These organs were closely related to AD, and* ATR* was closely related to these organs in the meridian theory.

(4) KEGG pathway annotation analysis showed that the potential targets of the compounds were clustered in 12 signaling pathways, mainly related to inflammation, calcium signaling pathway, G-protein mediated signaling pathway, and energy metabolism, and effects on the nervous system are mainly manifested in the dopaminergic synapse, cholinergic synapse, neurotrophin signaling pathway, serotonergic synapse, and LTP. Finally, pathway-mapping result showed that 9 protein targets were closely related to the* ATR* treatment AD.

In summary, this study not only conducts a comprehensive analysis of relevant AD through systematic pharmacological methods and discovers potential active compounds but also explains the treating mechanism of* ATR* in Alzheimer's disease, widely applied to clarify the effectiveness and mystery of the medicinal plant* ATR*; what is more, it provides an example for the treatment of complex diseases in the near future. Although the results are interesting, further research requires that the support of experimental data includes the confirmation of drug dose relationships. In addition, the present study needs to test the molecular mechanisms of the active compounds* in vivo* to support further assessment of potential clinical applications.

## Figures and Tables

**Table 1:** 32 potential active compounds of *Acori Tatarinowii Rhizoma* (*ATR*).

ID	Active compounds	OB	BBB	DL	Structure
M1	(-)-Alloaromadendrene	54.04	2.07	0.1	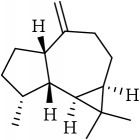
M2	(-)-*α*-Cedrene	55.56	2.16	0.1	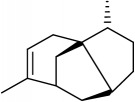
M3	(+)-*α*-Longipinene	57.47	2.05	0.12	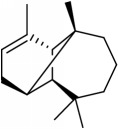
M4	(-)-Caryophyllene oxide	32.67	1.76	0.13	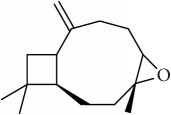
M5	(+)-11-Epispathulenol	81.61	1.55	0.12	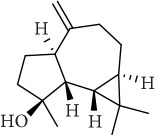
M6	(+)-Ledene	51.84	2.16	0.1	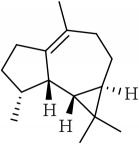
M7	(+)-Veraguensin	25.53	0.72	0.39	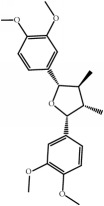
M8	2′-O-Methylisoliquiritigenin	75.86	-0.16	0.17	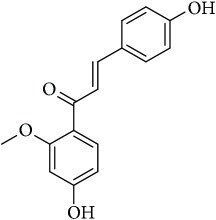
M9	Aminacrine	35	0.33	0.12	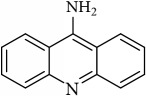
M10	*α*-Cubebene	16.73	2.1	0.11	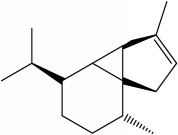
M11	Aristolone	45.31	1.54	0.13	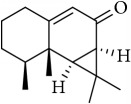
M12	Bergapten	42.21	0.69	0.13	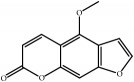
M13	*β*-Asarone	35.61	1.24	0.06	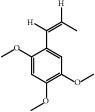
M14	*β*-Cubebene	32.81	2.02	0.11	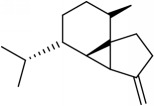
M15	*β*-Gurjunene	51.36	2.07	0.1	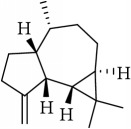
M16	*β*-Humulene	26.87	2.01	0.06	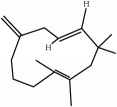
M17	Bisasarcin	18.55	0.54	0.5	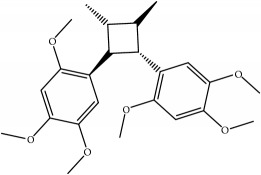
M18	Bisasaricin	28.94	0.65	0.5	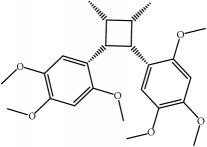
M19	Calamendiol	61.13	0.67	0.11	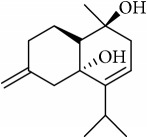
M20	Calarene	52.16	2.04	0.11	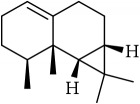
M21	cis-Methylisoeugenol	74.21	1.48	0.04	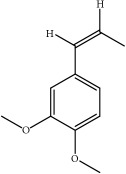
M22	Cycloartenol	38.69	1.33	0.78	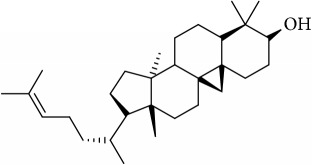
M23	Eudesmin	52.35	0.05	0.62	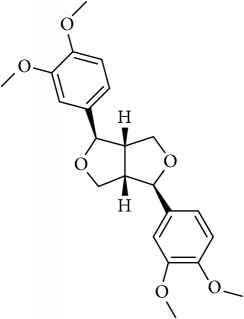
M24	*γ*-Asarone	22.76	1.33	0.06	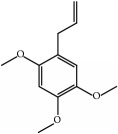
M25	Isocalamendiol	57.63	0.74	0.11	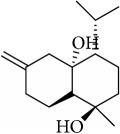
M26	Isopimpinellin	25.93	0.5	0.17	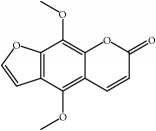
M27	Longicyclene	46.07	2.16	0.15	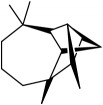
M28	Marmesin	50.28	0.07	0.18	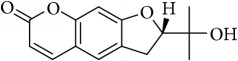
M29	Methyleugenol	73.36	1.41	0.04	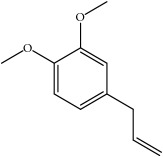
M30	Murolan-3, 9(11)-diene-10-peroxy	36.72	1.04	0.11	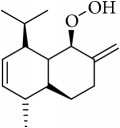
M31	Patchoulene	49.06	2.17	0.11	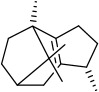
M32	*α*-Panasinsene	56.77	2.11	0.12	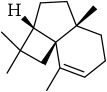

## Data Availability

The data used to support the findings of this study are available from the corresponding author upon request.

## Supplementary Materials

Supplementary Materials Table S1 shows the structure of 105 compounds in* Acori Tatarinowii Rhizoma* (ShiChangPu,* ATR*). Compounds information was found in the TCMSP (http://lsp.nwu.edu.cn/) and was input to Pubchem (https://www.ncbi.nlm.nih.gov/pccompound/) for compounds names to be uniformly standardized. Table S2 shows the targets prediction of 32 identified active compounds of* ATR*. Molecular targets information was found in the TCMSP (http://lsp.nwu.edu.cn/), SEA (http://sea.bkslab.org) and the Binding Database (http://www.bindingdb.org). The targets were obtained from different databases and input to UniProt (http://www.uniprot.org/) to make the targets names uniformly standardized. Noise and error information was eliminated by PharGKB (https://www.pharmgkb.org/), Therapeutic Target Database (DTT, http://bidd.nus.edu.sg/group/cjttd/), and the Comparative Toxicogenomics Database (CTD, http://ctdbase.org/). Table S3 shows the potential targets of 32 identified active compounds of* ATR* were associated with AD. In order to elucidate the action relations of* ATR* in Alzheimer's disease, the integration was performed by means of gene and AD vocabulary mapping by using the DisGeNET association type ontology to select the targets related to AD. Table S4 shows the 175 targets mRNA expression profiles in 84 normal tissues. The targets organ location network was used with the dataset: GeneAtlas U133A, gcrma (http://biogps.org).

## References

[1] Chinthapalli K. (2014). Alzheimer's disease: still a perplexing problem. *BMJ*.

[2] Alzheimers Dement (2016). 2016 Alzheimer’s disease facts and figures. *Alzheimer’s & Dementia: The Journal of the Alzheimer’s Association*.

[3] Burns A., Iliffe S. (2009). Alzheimer's disease. *British Medical Journal*.

[4] Ballard C., Gauthier S., Corbett A., Brayne C., Aarsland D., Jones E. (2011). Alzheimer's disease. *The Lancet*.

[5] Bhardwaj D., Mitra C., Narasimhulu C. A., Riad A., Doomra M., Parthasarathy S. (2017). Alzheimer's Disease - Current Status and Future Directions. *Journal of Medicinal Food*.

[6] Walker D., Lue L.-F. (2007). Anti-inflammatory and immune therapy for Alzheimer's disease: Current status and future directions. *Current Neuropharmacology*.

[7] Kitano H. H. (2007). A robustness-based approach to systems-oriented drug design. *Nature Reviews Drug Discovery*.

[8] Wang Y., Zheng C., Huang C. (2015). Systems Pharmacology Dissecting Holistic Medicine for Treatment of Complex Diseases: An Example Using Cardiocerebrovascular Diseases Treated by TCM. *Evidence-Based Complementary and Alternative Medicine*.

[9] Deng M.-Z., Huang L.-P., Fang Y.-Q. (2015). Effects of Total Ginsenosides and Volatile Oil of Acorus tatarinowii Co-Administration on Ability of Learning and Memory and Apoptosis in Alzheimer's Disease Mice Model Induced By D-Galactose and Aluminium Chloride. *Zhong yao cai = Zhongyaocai = Journal of Chinese Medicinal Materials*.

[10] Mao J., Huang S., Liu S. (2015). A herbal medicine for Alzheimer's disease and its active constituents promote neural progenitor proliferation. *Aging Cell*.

[11] An H. M., Li G. W., Lin C. (2014). Acorus tatarinowii Schott extract protects PC12 cells from amyloid-beta induced neurotoxicity. *Die Pharmazie*.

[12] Yang Y., Xuan L., Chen H. (2017). Neuroprotective Effects and Mechanism of. *Evidence-Based Complementary and Alternative Medicine*.

[13] Chang W., Teng J. (2015). *β*-asarone prevents A*β*25-35-induced inflammatory responses and autophagy in SH-SY5Y cells: Down expression Beclin-1, LC3B and up expression Bcl-2. *International Journal of Clinical and Experimental Medicine*.

[14] Lam K. Y. C., Chen J., Lam C. T. W. (2016). Asarone from Acori tatarinowii Rhizoma potentiates the nerve growth factor-induced neuronal differentiation in cultured PC12 cells: a signaling mediated by protein kinase A. *PLoS ONE*.

[15] Han T., Han P., Peng W., Wang X.-R. (2013). Antidepressant-like effects of essential oil and asarone, a major essential oil component from the rhizome of Acorus tatarinowii. *Pharmaceutical Biology*.

[16] Limón I. D., Mendieta L., Díaz A. (2009). Neuroprotective effect of alpha-asarone on spatial memory and nitric oxide levels in rats injected with amyloid-*β*(25-35). *Neuroscience Letters*.

[17] Liu W., Zhang B., Xin Z., Ren D., Yi L. (2017). GC-MS fingerprinting combined with chemometric methods reveals key bioactive components in Acori tatarinowii rhizoma. *International Journal of Molecular Sciences*.

[18] Nandakumar S., Menon S., Shailajan S. (2013). A rapid HPLC-ESI-MS/MS method for determination of *β*-asarone, a potential anti-epileptic agent, in plasma after oral administration of Acorus calamus extract to rats. *Biomedical Chromatography*.

[19] Wei G., Chen Y.-B., Chen D.-F. (2013). *β*-Asarone inhibits neuronal apoptosis via the CaMKII/CREB/Bcl-2 signaling pathway in an in vitro model and A*β*PP/PS1 mice. *Journal of Alzheimer's Disease*.

[20] Fang J., Liu C., Wang Q., Lin P., Cheng F. (2017). In silico polypharmacology of natural products. *Briefings in Bioinformatics*.

[21] Cai H., Luo Y., Yan X. (2018). The Mechanisms of Bushen-Yizhi Formula as a Therapeutic Agent against Alzheimer’s Disease. *Scientific Reports*.

[22] Luo Y., Wang Q., Zhang Y. (2016). A systems pharmacology approach to decipher the mechanism of danggui-shaoyao-san decoction for the treatment of neurodegenerative diseases. *Journal of Ethnopharmacology*.

[23] Wang L., Wu T., Yang C. (2017). Network pharmacology-based study on the mechanism of action for herbal medicines in Alzheimer treatment. *Journal of Ethnopharmacology*.

[24] Ru J., Li P., Wang J. (2014). TCMSP: a database of systems pharmacology for drug discovery from herbal medicines. *Journal of Cheminformatics*.

[25] Su X., Kong L., Lei X., Hu L., Ye M., Zou H. (2007). Biological fingerprinting analysis of traditional Chinese medicines with targeting ADME/Tox property for screening of bioactive compounds by chromatographic and MS methods. *Mini-Reviews in Medicinal Chemistry*.

[26] Xu X., Zhang W., Huang C. (2012). A novel chemometric method for the prediction of human oral bioavailability. *International Journal of Molecular Sciences*.

[27] Tang F., Tang Q., Tian Y., Fan Q., Huang Y., Tan X. (2015). Network pharmacology-based prediction of the active ingredients and potential targets of Mahuang Fuzi Xixin decoction for application to allergic rhinitis. *Journal of Ethnopharmacology*.

[28] Yangt F., Xu J., Zeng J. Drug-target interaction prediction by integrating chemical, genomic, functional and pharmacological data.

[29] Tattersall M., Sodergren J. E., Sengupta S. K., Trites D. H., Modest E. J., Frei E. (1975). Pharmacokinetics of actinomycin 0 in patients with malignant melanoma. *Clinical Pharmacology & Therapeutics*.

[30] Keiser M. J., Roth B. L., Armbruster B. N., Ernsberger P., Irwin J. J., Shoichet B. K. (2007). Relating protein pharmacology by ligand chemistry. *Nature Biotechnology*.

[31] Chen X., Liu M., Gilson M. K. (2001). BindingDB: A web-accessible molecular recognition database. *Combinatorial Chemistry & High Throughput Screening*.

[32] Wu C. H., Apweiler R., Bairoch A. (2006). The Universal Protein Resource (UniProt): an expanding universe of protein information. *Nucleic Acids Research*.

[33] Altman R. B. (2007). Pharmgkb: a logical home for knowledge relating genotype to drug response phenotype. *Nature Genetics*.

[34] Chen X., Ji Z. L., Chen Y. Z. (2002). TTD: therapeutic target database. *Nucleic Acids Research*.

[35] Davis A. P., Grondin C. J., Johnson R. J. (2017). The Comparative Toxicogenomics Database: Update 2017. *Nucleic Acids Research*.

[36] Shannon P., Markiel A., Ozier O. (2003). Cytoscape: a software Environment for integrated models of biomolecular interaction networks. *Genome Research*.

[37] Piñero J., Bravo Á., Queralt-Rosinach N. (2017). DisGeNET: A comprehensive platform integrating information on human disease-associated genes and variants. *Nucleic Acids Research*.

[38] Wu C., Orozco C., Boyer J. (2009). BioGPS: an extensible and customizable portal for querying and organizing gene annotation resources. *Genome Biology*.

[39] Wu C., Jin X., Tsueng G., Afrasiabi C., Su A. I. (2016). BioGPS: Building your own mash-up of gene annotations and expression profiles. *Nucleic Acids Research*.

[40] Ashburner M., Ball C. A., Blake J. A. (2000). Gene ontology: tool for the unification of biology. *Nature Genetics*.

[41] Huang D. W., Sherman B. T., Lempicki R. A. (2009). Systematic and integrative analysis of large gene lists using DAVID bioinformatics resources. *Nature Protocols*.

[42] Du J. L., Yuan Z. F., Ma Z. W., Song J. Z., Xie X. L., Chen Y. L. (2014). KEGG-PATH: kyoto encyclopedia of genes and genomes-based pathway analysis using a path analysis model. *Molecular BioSystems*.

[43] Ogata H., Goto S., Sato K., Fujibuchi W., Bono H., Kanehisa M. (1999). KEGG: kyoto encyclopedia of genes and genomes. *Nucleic Acids Research*.

[44] Liu X. C., Zhou L. G., Liu Z. L., Du S. S. (2013). Identification of insecticidal constituents of the essential oil of Acorus calamus rhizomes against Liposcelis bostrychophila badonnel. *Molecules*.

[45] Choi Y. K., Cho G.-S., Hwang S. (2010). Methyleugenol reduces cerebral ischemic injury by suppression of oxidative injury and inflammation. *Free Radical Research*.

[46] Lam K. Y. C., Yao P., Wang H., Duan R., Dong T. T. X., Tsim K. W. K. (2017). Asarone from Acori Tatarinowii Rhizome prevents oxidative stress-induced cell injury in cultured astrocytes: A signaling triggered by Akt activation. *PLoS ONE*.

[47] Cho J. Y., Yoo E. S., Baik K. U., Park M. H. (1999). Eudesmin inhibits tumor necrosis factor-*α* production and T cell proliferation. *Archives of Pharmacal Research*.

[48] Liu H., Song Z., Liao D.-G. (2015). Anticonvulsant and Sedative Effects of Eudesmin isolated from Acorus tatarinowii on mice and rats. *Phytotherapy Research*.

[49] Kim J. H., Kim M. S., Lee B. H. (2017). Marmesin-mediated suppression of VEGF/VEGFR and integrin *β*1 expression: Its implication in non-small cell lung cancer cell responses and tumor angiogenesis. *Oncology Reports*.

[50] Liang J., Wu J., Liu Y. (2017). Patchoulene Epoxide Isolated from Patchouli Oil Suppresses Acute Inflammation through Inhibition of NF-*κ*B and Downregulation of COX-2/iNOS. *Mediators of Inflammation*.

[51] Zhou Y., Wang J., Yang W. (2017). Bergapten prevents lipopolysaccharide-induced inflammation in RAW264.7 cells through suppressing JAK/STAT activation and ROS production and increases the survival rate of mice after LPS challenge. *International Immunopharmacology*.

[52] Fang K., Dong H., Jiang S. (2016). Diosgenin and 5-Methoxypsoralen Ameliorate Insulin Resistance through ER--*α*/PI3K/Akt-Signaling Pathways in HepG2 Cells. *Evidence-Based Complementary and Alternative Medicine*.

[53] Walker T. M., Atterwill C., Dewhurst B. B. (1995). Potential neurotoxicity of a novel aminoacridine analogue. *Human & Experimental Toxicology*.

[54] Johnston W. H., Karchesy J. J., Constantine G. H., Craig A. M. (2001). Antimicrobial activity of some Pacific Northwest woods against anaerobic bacteria and yeast. *Phytotherapy Research*.

[55] da Silva Filho A. A., Andrade Silva M. L., Carvalho J. C. T., Bastos J. K. (2004). Evaluation of analgesic and anti-inflammatory activities of Nectandra megapotamica (Lauraceae) in mice and rats. *Journal of Pharmacy and Pharmacology*.

[56] Zhai H., Inoue T., Moriyama M., Esumi T., Mitsumoto Y., Fukuyama Y. (2005). Neuroprotective effects of 2,5-diaryl-3,4-dimethyltetrahydrofuran neolignans. *Biological & Pharmaceutical Bulletin*.

[57] Huang L., Deng M., Zhang S., Lu S., Gui X., Fang Y. (2017). *β*-asarone and levodopa coadministration increases striatal levels of dopamine and levodopa and improves behavioral competence in Parkinson's rat by enhancing dopa decarboxylase activity. *Biomedicine & Pharmacotherapy*.

[58] Marrelli M., Cristaldi B., Menichini F., Conforti F. (2015). Inhibitory effects of wild dietary plants on lipid peroxidation and on the proliferation of human cancer cells. *Food and Chemical Toxicology*.

[59] El-Soud N. H. A., Deabes M., El-Kassem L. A., Khalil M. (2015). Chemical composition and antifungal activity of ocimum basilicum L. essential oil. *Macedonian Journal of Medical Sciences*.

[60] Thusoo S., Gupta S., Sudan R. (2014). Antioxidant activity of essential oil and extracts of *Valeriana jatamansi*roots. *BioMed Research International*.

[61] Li D., Wu L. (2017). Coumarins from the roots of angelica dahurica cause anti-allergic inflammation. *Experimental and Therapeutic Medicine*.

[62] Manoharan R. K., Lee J.-H., Kim Y.-G., Kim S.-I., Lee J. (2017). Inhibitory effects of the essential oils *α*-longipinene and linalool on biofilm formation and hyphal growth of Candida albicans. *Biofouling*.

[63] Juárez Z. N., Bach H., Sánchez-Arreola E., Bach H., Hernández L. R. (2016). Protective antifungal activity of essential oils extracted from Buddleja perfoliata and Pelargonium graveolens against fungi isolated from stored grains. *Journal of Applied Microbiology*.

[64] Lin J., Cai Q.-Y., Xu W., Lin J.-M., Peng J. (2016). Chemical composition, anticancer, anti-neuroinflammatory, and antioxidant activities of the essential oil of Patrinia scabiosaefolia. *Chinese Journal of Integrative Medicine*.

[65] Yu H., Chen J., Xu X. (2012). A systematic prediction of multiple drug-target interactions from chemical, genomic, and pharmacological data. *PLoS ONE*.

[66] Liu J., Pei T., Mu J. (2016). Systems Pharmacology Uncovers the Multiple Mechanisms of Xijiao Dihuang Decoction for the Treatment of Viral Hemorrhagic Fever. *Evidence-Based Complementary and Alternative Medicine*.

[67] Berger S. I., Iyengar R. (2009). Network analyses in systems pharmacology. *Bioinformatics*.

[68] Jeong H., Mason S. P., Barabási A. L., Oltvai Z. N. (2001). Lethality and centrality in protein networks. *Nature*.

[69] Yang H., Zhang W., Huang C. (2014). A novel systems pharmacology model for herbal medicine injection: A case using reduning injection. *BMC Complementary and Alternative Medicine*.

[70] Zhang J., Li Y., Chen S.-S. (2015). Systems pharmacology dissection of the anti-inflammatory mechanism for the medicinal herb *Folium eriobotryae*. *International Journal of Molecular Sciences*.

[71] Pan J.-B., Hu S.-C., Shi D. (2013). PaGenBase: a pattern gene database for the global and dynamic understanding of gene function. *PLoS ONE*.

[72] Zhang W., Tao Q., Guo Z. (2016). Systems pharmacology dissection of the integrated treatment for cardiovascular and gastrointestinal disorders by traditional chinese medicine. *Scientific Reports*.

[73] Franceschi M., Ferini-Strambi L., Minicucci F., Sferrazza-Papa A., Smirne S. (1986). Signs of cardiac autonomic dysfunction during sleep in patients with Alzheimer's disease. *Gerontology*.

[74] Nilsson K., Gustafson L., Hultberg B. (2004). Plasma homocysteine concentration relates to the severity but not to the duration of Alzheimer's disease. *International Journal of Geriatric Psychiatry*.

[75] De La Monte S. M., Wands J. R. (2005). Review of insulin and insulin-like growth factor expression, signaling, and malfunction in the central nervous system: Relevance to Alzheimer's disease. *Journal of Alzheimer's Disease*.

[76] Lee T. A., Wolozin B., Weiss K. B., Bednar M. M. (2005). Assessment of the emergence of Alzheimer's disease following coronary artery bypass graft surgery or percutaneous transluminal coronary angioplasty. *Journal of Alzheimer's Disease*.

[77] Palotás A., Reis H. J., Bogáts G. (2010). Coronary artery bypass surgery provokes alzheimer's disease-like changes in the cerebrospinal fluid. *Journal of Alzheimer's Disease*.

[78] Glass C. K., Saijo K. (2010). Nuclear receptor transrepression pathways that regulate inflammation in macrophages and T cells. *Nature Reviews Immunology*.

[79] Serhan C. N. (2008). Controlling the resolution of acute inflammation: A new genus of dual anti-inflammatory and proresolving mediators. *Journal of Periodontology*.

[80] Man H.-Y., Sekine-Aizawa Y., Huganir R. L. (2007). Regulation of *α*-amino-3-hydroxy-5-methyl-4-isoxazolepropionic acid receptor trafficking through PKA phosphorylation of the Glu receptor 1 subunit. *Proceedings of the National Acadamy of Sciences of the United States of America*.

[81] Sun L., Wang X., Liu S. (2005). Bilateral injection of isoproterenol into hippocampus induces Alzheimer-like hyperphosphorylation of tau and spatial memory deficit in rat. *FEBS Letters*.

[82] Clapham D. E. (2007). Calcium Signaling. *Cell*.

[83] Ivannikov M. V., Sugimori M., Llinás R. R. (2013). Synaptic vesicle exocytosis in hippocampal synaptosomes correlates directly with total mitochondrial volume. *Journal of Molecular Neuroscience*.

[84] Berridge M. J. (1998). Neuronal calcium signaling. *Neuron*.

[85] Laura P., Sandra L.-G., Jérôme G. (2013). Rab4b controls an early endosome sorting event by interacting with the c-Subunit of the clathrin adaptor complex 1. *Journal of Cell Science*.

[86] Peltier J., O'Neill A., Schaffer D. V. (2007). PI3K/Akt and CREB regulate adult neural hippocampal progenitor proliferation and differentiation. *Developmental Neurobiology*.

[87] Walker C. L., Walker M. J., Liu N.-K. (2012). Systemic bisperoxovanadium activates Akt/mTOR, reduces autophagy, and enhances recovery following cervical spinal cord injury. *PLoS ONE*.

[88] Qi Y., Dou D.-Q., Jiang H. (2017). Arctigenin Attenuates Learning and Memory Deficits through PI3k/Akt/GSK-3 *β* Pathway Reducing Tau Hyperphosphorylation in A *β* -Induced AD Mice. *Planta Medica*.

[89] Man H.-Y., Wang Q., Lu W.-Y. (2003). Activation of PI3-kinase is required for AMPA receptor insertion during LTP of mEPSCs in cultured hippocampal neurons. *Neuron*.

[90] Yan R., Fan Q., Zhou J., Vassar R. (2016). Inhibiting BACE1 to reverse synaptic dysfunctions in Alzheimer's disease. *Neuroscience & Biobehavioral Reviews*.

[91] Ferreira-Vieira T. H., Guimaraes I. M., Silva F. R., Ribeiro F. M. (2016). Alzheimer's disease: Targeting the cholinergic system. *Current Neuropharmacology*.

[92] Kamkwalala A. R., Newhouse P. A. (2017). Beyond acetylcholinesterase inhibitors: Novel cholinergic treatments for Alzheimer’s disease. *Current Alzheimer Research*.

[93] Ledo J. H., Azevedo E. P., Beckman D. (2016). Cross talk between brain innate immunity and serotonin signaling underlies depressive-like behavior induced by Alzheimer’s amyloid-*β* oligomers in mice. *The Journal of Neuroscience*.

[94] Shankar G. M., Li S., Mehta T. H. (2008). Amyloid-*β* protein dimers isolated directly from Alzheimer's brains impair synaptic plasticity and memory. *Nature Medicine*.

[95] Hardy J., Selkoe D. J. (2002). The amyloid hypothesis of Alzheimer's disease: progress and problems on the road to therapeutics. *Science*.

[96] Kennedy M. E., Stamford A. W., Chen X. (2016). The BACE1 inhibitor verubecestat (MK-8931) reduces CNS b-Amyloid in animal models and in Alzheimer's disease patients. *Science Translational Medicine*.

[97] Sims J. R., Selzler K. J., Downing A. M. (2017). Development Review of the BACE1 Inhibitor Lanabecestat (AZD3293/LY3314814). *The Journal of Prevention of Alzheimer's Disease*.

[98] Mudher A., Lovestone S. (2002). Alzheimer's disease -do tauists and baptists finally shake hands?. *Trends in Neurosciences*.

[99] Nicoletti F., Bruno V., Copani A., Casabona G., Knöpfel T. (1996). Metabotropic glutamate receptors: A new target for the therapy of neurodegenerative disorders?. *Trends in Neurosciences*.

[100] Lipton S. A. (2006). Paradigm shift in neuroprotection by NMDA receptor blockade: memantine and beyond. *Nature Reviews Drug Discovery*.

[101] Hardingham G. E., Bading H. (2010). Synaptic versus extrasynaptic NMDA receptor signalling: implications for neurodegenerative disorders. *Nature Reviews Neuroscience*.

[102] Huang C., Zheng C., Li Y., Wang Y., Lu A., Yang L. (2014). Systems pharmacology in drug discovery and therapeutic insight for herbal medicines. *Briefings in Bioinformatics*.

